# Glandular defects in the mouse uterus with sustained activation of TGF-beta signaling is associated with altered differentiation of endometrial stromal cells and formation of stromal compartment

**DOI:** 10.1371/journal.pone.0209417

**Published:** 2018-12-14

**Authors:** Nan Ni, Yang Gao, Xin Fang, Maria Melgar, David F. Vincent, John P. Lydon, Laurent Bartholin, Qinglei Li

**Affiliations:** 1 Department of Veterinary Integrative Biosciences, Texas A&M University, College Station, Texas, United States of America; 2 Cancer Research United Kingdom Beatson Institute, Garscube Estate, Glasgow, United Kingdom; 3 Department of Molecular and Cellular Biology, Baylor College of Medicine, Houston, Texas, United States of America; 4 Centre de Recherche en Cancérologie de Lyon, INSERM U1052, CNRS UMR5286, Université Lyon 1, Centre Léon Bérard, Lyon, France; Universite du Quebec a Trois-Rivieres, CANADA

## Abstract

Uterine gland development, also known as adenogenesis, is a key uterine morphogenic process indispensable for normal uterine function and fertility. Our earlier studies have reported that overactivation of TGFB receptor 1 (TGFBR1) in the mouse uterus using progesterone receptor (*Pgr*)-Cre recombinase causes female infertility, defective decidualization, and reduced uterine gland formation, a developmental milestone of postnatal uterus. To understand mechanisms that underpin the disrupted uterine gland formation in mice with sustained activation of TGFBR1, we raised the question of whether early postnatal adenogenesis was compromised in these mice. Experiments were designed using mice with constitutive activation of TGFBR1 driven by *Pgr*-Cre to determine the timing of adenogenic defects and potential mechanisms associated with dysregulation of adenogenic genes, luminal epithelial cell proliferation and endometrial fibrotic changes. Uterine tissues from mice with constitutive activation of TGFBR1 were collected during the critical time window of adenogenesis and analyzed together with age-matched controls. Multiple approaches including immunohistochemistry, immunofluorescence, Trichrome staining, quantitative real-time PCR, western blot, conditional knockout and human endometrial cell culture were utilized. TGFBR1 activation in the mouse uterus suppressed adenogenesis during postnatal uterine development, concomitant with the aberrant differentiation of uterine stromal cells. Analysis of transcript expression of WNT pathway components revealed dysregulation of adenogenesis-associated genes. Notably, the adenogenic defects occurred in spite of the increased proliferation of uterine luminal epithelial cells, accompanied by increased expression of genes associated with fibrotic changes. Moreover, the adenogenic defects were alleviated in mice where TGFBR1 was activated in presumably half of the complement of uterine cells. Our results suggest that altered differentiation of endometrial stromal cells and formation of stromal compartment promote adenogenic defects.

## Introduction

An increasing number of reproductive-aged women face pregnancy loss and infertility, some of which are associated with uterine dysfunction. Transforming growth factor beta (TGFB) superfamily members are evolutionarily conserved and fundamental regulators of cell growth and differentiation. Critical roles of TGFB superfamily members in female reproduction including post-implantation uterine function and pregnancy maintenance have been demonstrated, with the application of genetically engineered mouse models [1–3].

TGFB ligands (TGFBs1-3) signal through a receptor complex consisting of TGFB type 1 and type 2 receptors (TGFBR1/TGFBR2). Canonically, activated receptors impinge on receptor-regulated SMADs (R-SMADs) and SMAD4, the common SMAD, to elicit biological responses in target cells through the regulation of gene transcription [4]. The *in vivo* function of TGFB signaling in the uterus remains incompletely understood [5–9]. Recent advances in tissue/cell specific targeting technology using Cre-LoxP system have been effective in deciphering gene function in reproduction and development [1, 3, 10, 11].

By taking advantage of TGFBR1 conditional loss-of-function and gain-of-function mouse models, we have gained a new understanding of TGFB signaling in female reproductive tract development and function [3, 12, 13]. Because balanced TGFB signaling controls homeostatic cellular processes, our approach to use both loss-of-function and gain-of-function mouse models is complementary and beneficial to define the role of TGFB signaling in both physiologic and pathologic conditions. In an earlier report, we generated a mouse model harboring a constitutively active *TGFBR1* allele, the expression of which was conditionally driven by the progesterone receptor (*Pgr*)-Cre recombinase [13]. These mice were sterile and developed several phenotypic abnormalities including enlarged myometrial compartment, disorganized myometrium, reduced stromal compartment and impaired uterine gland formation [13].

Uterine gland development, also known as adenogenesis, is a key uterine morphogenic process indispensable for normal uterine function [14–18]. Recent studies from the Spencer laboratory showed that uterine glands play critical roles in embryo implantation and endometrial decidualization, essential events for a successful pregnancy [19]. The mechanisms underlying the development of uterine glands are not well delineated. Adenogenesis is a complex physiological event whereby a number of genes, including, but not limited to forkhead box A2 (*Foxa2*), wingless-type MMTV integration site family member 4 (*Wnt4*), *Wnt5a*, *Wnt7a* and E-cadherin (*Cdh1*), are known to be essential [18, 20–27]. Conditional deletion of FOXA2, a uterine gland-specific transcription factor, causes a marked reduction in the number of uterine glands [18, 20]. WNT signaling plays critical roles in adenogenesis. For example, deletion of *Wnt7a* or *Wnt4* in the mouse uterus results in lack of uterine glands or reduced number of uterine glands [21, 25]. Conditional ablation of CDH1, a cell-cell adhesion molecule, leads to loss of uterine glands in the neonatal uterus [26]. Hormone-related activities are also known to affect adenogenesis. Chronic exposure to progestin in ewes from birth blocks adenogenesis, leading to ablation of endometrial glands [28]. Progesterone treatment also suppresses uterine gland development in the neonatal mouse uterus, along with reduced proliferation of the luminal epithelial cells [29]. In addition, epithelial-mesenchymal interactions are important for female reproductive tract development [30], and its involvement in adenogenesis requires further investigation.

The role of TGFB signaling in the adenogenic process is poorly defined. Of note, adenogenic defects have not been reported in *Tgfbr1* conditionally ablated mice using *Pgr*-Cre [31, 32], indicating that TGFBR1 is not essential for adenogenesis. However, constitutively active TGFBR1 in the mouse uterus severely impaired uterine gland formation [13]. To understand potential mechanisms that underpin the disrupted uterine gland formation in mice with sustained activation of TGFBR1, we determined whether postnatal adenogenic process was compromised at histological, cellular, and molecular perspectives. Additionally, we generated a complementary mouse model, in which activation of TGFBR1 occurs presumably in half of the complement of uterine cells to further understand the effect of TGFBR1 activation on adenogenesis. Our results show that overactivation of TGFBR1 impairs the differentiation of endometrial stromal cells and the formation of an integral stromal compartment, resulting in adenogenic defects. This finding underscores the importance of stromal-epithelial interaction during uterine development.

## Materials and methods

### Ethics statement

Mice were housed in the Texas A&M University Laboratory Animal Resources and Research (LARR) facility from the Comparative Medicine Program under a 12-hour light, 12-hour dark cycle and had access to the food and water ad libitum. Mice were cared by experienced veterinary technicians and trained research staff. Animal use protocol for this study was approved by the Institutional Animal Care and Use Committee (IACUC) at Texas A&M University (protocol numbers: 2014–0346 & 2016–0198). Animal manipulation and handling were performed according to the Guide for the Care and Use of Laboratory Animals guideline of National Institute of Health and Texas A&M IACUC.

### Animals

*TGFBR1*^CA^ flox allele was constructed by targeting the constitutively active TGFBR1 into hypoxanthine phosphoribosyl-transferase (*Hprt*) locus, and the resultant mice harbor a functional *Hprt* gene [33]. Generation of *Pgr*-Cre mice and *TGFBR1*^CA flox/flox^; *Pgr*^Cre/+^ mice was detailed elsewhere [13, 32]. To generate *TGFBR1*^CA flox/+^; *Pgr*^Cre/+^ mice, the *TGFBR1*^CA flox/flox^ mice were bred with *Pgr*^Cre/+^ mice. Mice were genotyped by genomic polymerase chain reaction (PCR) to determine the presence of *TGFBR1*^CA^ flox allele and *Pgr*-Cre using specific primers reported previously [1, 33].

### Sample preparation

Uterine samples were collected from control and experimental groups at different timepoints including D5, D7, D15, D21 and D31 and fixed in 10% neutral buffered formalin for immunohistochemistry and immunofluorescence analysis or homogenized in lysis buffer supplemented by RNeasy Mini Kit (Qiagen) and stored at −80°C until use.

### Immunofluorescence and immunohistochemistry

Tissue processing and embedding were carried out using the histology core facility of the Department of Veterinary Integrative Biosciences at Texas A&M University. Paraffin sections (5 μm) were used for both immunofluorescence and immunohistochemistry [3, 12]. Briefly, sections were deparaffinized in xylene and rehydrated in graded ethanol. Then the slides were subject to antigen retrieval using 10 mM citrate buffer (pH 6.0). For immunofluorescence microscopy, sections were blocked with 5% bovine serum albumin (BSA) and incubated at 4°C overnight with antibodies including mouse anti-alpha smooth muscle actin (ACTA2; ab76549; 1:1600; Abcam), rat anti-cytokeratin 8 (KRT8) (1:100; TROMA-I; Developmental Studies Hybridoma Bank), rabbit anti- FOXA2 (1:250; ab108422; Abcam), goat anti-forkhead box L2 (FOXL2; ab5096; 1:1500; Abcam) and rabbit anti-antigen identified by monoclonal antibody Ki 67 (Ki67; ab16667; 1:200; Abcam). Subsequent incubation of the sections with secondary antibodies conjugated with Alexa Fluor 488 or 594 (1:400; Invitrogen) was performed at room temperature (RT) for 1 h. The slides were mounted using ProLong Gold Slowfade media with DAPI (Invitrogen). Immunofluorescence signals were examined under an IX73 microscope interfaced with an XM10 CCD camera and cellSens Digital Imaging Software (Olympus). For immunohistochemical analysis, sections following antigen retrieval were treated with 3% H_2_O_2_ to quench endogenous peroxidase activity. Then, the sections were blocked with 5% non-immune serum and sequentially incubated with primary antibodies including rat anti-KRT8 (1:200; TROMA-I; Developmental Studies Hybridoma Bank), rabbit anti-estrogen receptor α (ERα; sc-542; 1:1000; Santa Cruz), rabbit anti-PGR (1:50; MA5-14505; Thermo Scientific), rabbit anti-calponin (CNN1; 1:500; #04589; EMD Millipore), rabbit anti-vimentin (VIM; 1:200; #5741; Cell Signaling Technology) and rabbit anti-collagen I (COL-1; 1:200; ab34710; Abcam), secondary antibody and Avidin/Biotin Complex (ABC; Vector Laboratories). The signals were developed using NovaRED Peroxidase Substrate Kit (Vector Laboratories). The sections were counterstained with hematoxylin and mounted with Permount (Fisher Scientific). Isotype-matched IgGs were included as negative controls.

### Trichrome staining

Trichrome Staining was performed using Trichrome Stain Kit from Abcam (ab150686) based on manufacturer’s instructions. In brief, serial paraffin sections were deparaffinized and rehydrated. Post fixation was conducted using Bouin’s Fluid for 60 min. Slides were then rinsed and incubated with equal volumes of Weigert’s A and Weigert’s B Iron hematoxylin for 5 min. After being washed, slides were sequentially incubated with Biebrich Scarlet/Acid Fuchsin Solution and Phosphomolybdic/ Phosphotungstic Acid for 3 and 15 min, respectively. Slides were stained with Aniline Blue Solution for 10 min, followed by 3 min incubation with Acetic Acid Solution (1%). Then, slides were dehydrated and mounted.

### Human endometrial stromal cell culture and treatment

Human endometrial stromal cells (T-HESC; ATCC No. CRL-4003) [34] were cultured in phenol red-free DMEM/F12 supplemented with 10% charcoal-stripped fetal bovine serum (FBS; HyClone), 1% ITS + Premix (BD), 500 ng/ml puromycin (Invitrogen) and 100 U/ml Penicillin and 100 μg/ml Streptomycin. Cells were maintained at 37°C supplemented with 5% CO2. Authentication of cells was performed by ATCC using short tandem repeat (STR) analysis. The cells were further verified using a functional assay to demonstrate their ability to undergo decidualization response via increasing the expression of insulin like growth factor binding protein 1 (*IGFBP1*) mRNA upon treatment with 8-bromoadenosine 3', 5'-cyclic monophosphate (8-Br-cAMP) (0.5 mM) (S1 Fig). For TGFB1 treatment, the cells were serum-starved overnight and then treated with vehicle (VEHL) or TGFB1 (0.1–10 ng/ml; R&D) for 24 h. Cells were collected and total RNA and proteins isolated as described below.

### RNA isolation, reverse-transcription, and real-time PCR

Total RNA was extracted using RNeasy Mini Kit (Qiagen) according to the manufacturer’s instruction, with on-column DNase digestion performed to eliminate potential DNA contamination. RNA was dissolved in ribonuclease-free water and quantified using a NanoDrop Spectrophotometer ND 1000 (NanoDrop Technologies).

Reverse transcription (RT) and real-time RT-PCR analysis using CFX Connect real-time PCR Detection System (Bio-Rad) and SYBR green were conducted as described [12]. The assays were performed in duplicate for each sample. Primers were synthesized based on sequences from published reports [17, 26, 35–39], PrimerBank and RTPrimerDB [40, 41] (Table 1). TaqMan gene expression assays were conducted using TaqMan probes for *Foxa2* (Mm01976556_s1), WAP four-disulfide core domain 3 (*Wfdc3*; Mm01243777_m1), chemokine (C-X-C motif) ligand 15 (*Cxcl15*; Mm00441263_m1), ribosomal protein L19 (*Rpl19*; Mm02601633_g1), *IGFBP1* (Hs00236877_m1), integrin subunit alpha 1 (*ITGA1*; Hs00235006_m1), collagen type I alpha 1 chain (*COL1A1*; Hs00164004_m1) and *RPL19* (Hs02338565_gH) based on manufacturer’s instruction (Thermo Scientific). The average cycle threshold (CT) values were calculated and DDCT method was used to determine relative gene expression levels [42].

**Table 1:** Sequences of real-time RT-PCR primers.

Name	Sequence (5′-3′)		Reference
*Col1a1*	Forward	GCTCCTCTTAGGGGCCACT	PrimerBank ID 34328108a1
	Reverse	CCACGTCTCACCATTGGGG	
*Itga1*	Forward	CCTTCCCTCGGATGTGAGTCA	PrimerBank ID 26343427a1
	Reverse	AAGTTCTCCCCGTATGGTAAGA	
*Itgb1*	Forward	ATGCCAAATCTTGCGGAGAAT	PrimerBank ID 52722a1
	Reverse	TTTGCTGCGATTGGTGACATT	
*Lama1*	Forward	CGGGTCTGTGACGGTAACAGT	[35]
	Reverse	GCCATCGATTGCGTGTGAT	
*Acta2*	Forward	GTCCCAGACATCAGGGAGTAA	PrimerBank ID 6671507a1
	Reverse	TCGGATACTTCAGCGTCAGGA	
*Wnt4*	Forward	CATCGAGGAGTGCCAATACCA	[26]
	Reverse	GGAGGGAGTCCAGTGTGGAA	
*Wnt5a*	Forward	GGCGAGCTGTCTACCTGTGG	[26]
	Reverse	GGCGAACGGGTGACCATAGT	
*Wnt7a*	Forward	CGACTGTGGCTGCGACAAG	RTPrimerDB ID 3399
	Reverse	CTTCATGTTCTCCTCCAGGATCTTC	
*Wnt11*	Forward	GCCATGAAGGCCTGCCGTAG	[26]
	Reverse	GATGGTGTGACTGATGGTGG	
*Wnt16*	Forward	CAGGGCAACTGGATGTGGTT	PrimerBank ID 26335741a1
	Reverse	CTAGGCAGCAGGTACGGTT	
*Fzd6*	Forward	GCGGCGTTTGCTTCGTT	[26]
	Reverse	CACAGAGGCAGAAGGACGAAGT	
*Fzd10*	Forward	TGCTCTGACCGGCTTCGT	[17]
	Reverse	GATGAAGGAAGTGCCGATGAC	
*Ctnnb1*	Forward	ATGGAGCCGGACAGAAAAGC	PrimerBank ID 6671684a1
	Reverse	CTTGCCACTCAGGGAAGGA	
*Sfrp1*	Forward	CAACGTGGGCTACAAGAAGAT	PrimerBank ID 7305481a1
	Reverse	GGCCAGTAGAAGCCGAAGAAC	
*Sfrp2*	Forward	CGTGGGCTCTTCCTCTTCG	[38]; Primerbank ID 6677895a1
	Reverse	ATGTTCTGGTACTCGATGCCG	
*Sfrp3*	Forward	ATTTGGTGTTCTGTACCCTG	[39]
	Reverse	CGTTTCCTCATAAAATGCTTC	
*Sfrp4*	Forward	AGAAGGTCCATACAGTGGGAAG	PrimerBank ID 7710094a1
	Reverse	GTTACTGCGACTGGTGCGA	
*Sfrp5*	Forward	CACTGCCACAAGTTCCCCC	PrimerBank ID 31560421a1
	Reverse	TCTGTTCCATGAGGCCATCAG	
*Foxl2*	Forward	GCTACCCCGAGCCCGAAGAC	[36]
	Reverse	GTGTTGTCCCGCCTCCCTTG	
*Vim*	Forward	GCTGCGAGAGAAATTGCAGGA	PrimerBank ID 227430362c3
	Reverse	CCACTTTCCGTTCAAGGTCAAG	
*Cd10*	Forward	CTCTCTGTGCTTGTCTTGCTC	PrimerBank ID 31543255a1
	Reverse	GACGTTGCGTTTCAACCAGC	
*ACTA2*	Forward	TCAATGTCCCAGCCATGTAT	[37]
	Reverse	CAGCACGATGCCAGTTGT	
*CTGF*	Forward	TTGGCCCAGACCCAACTATG	RTPrimerDB ID 596
	Reverse	CAGGAGGCGTTGTCATTGGT	

### Western blotting

Protein samples were prepared from T-HESCs using radioimmunoprecipitation assay (RIPA) buffer. Cell lysates were kept on ice for 30 mins, followed by sonication and centrifugation. Supernatants containing soluble proteins were quantified using bicinchoninic acid method (Thermo Fisher Scientific). Approximately 15 μg of proteins were loaded onto 12% Mini-PROTEAN TGX Precast Gels (Bio-Rad). After electrophoresis, proteins were transferred to polyvinylidene difluoride (PVDF) membranes by using Bio-Rad Trans-Blot Turbo Transfer System. The PVDF membranes were then blocked with 5% non-fat milk and incubated with rabbit anti-COL-1 (1:1000; ab34710; Abcam), rabbit anti- connective tissue growth factor (CTGF; 1:1000; ab6992; Abcam), rabbit anti-ACTA2 (1:4000; #19245; Cell Signaling Technology), mouse anti-ITGA1 (1:250; sc-271034; Santa Cruz) and rabbit anti-GAPDH (1:1000; # 2118; Cell Signaling Technology) overnight at 4°C. Then, membranes were washed and incubated with horseradish peroxidase (HRP)-conjugated antibody (1:20,000) for 1 h at room temperature. Following antibody incubation and extensive washes, Immobilon Western Chemiluminescent HRP Substrate (EMD Millipore) was added to the membranes for signal detection. Images were developed using a Bio-Rad ChemiDoc imaging system.

### Statistical analysis

Comparisons of two means were made using student *t*-test. One way analysis of variance (ANOVA) was used to determine significance among multiple groups followed by Tukey’s HSD. Data are shown as mean ± SEM. Significance was reported at *P < 0.05, **P < 0.01 and ***P < 0.001 or indicated using different letters (*P* < 0.05).

## Results

### Sustained activation of TGFB signaling in the uterus leads to defective adenogenesis at early postnatal development

**Fig 1:**
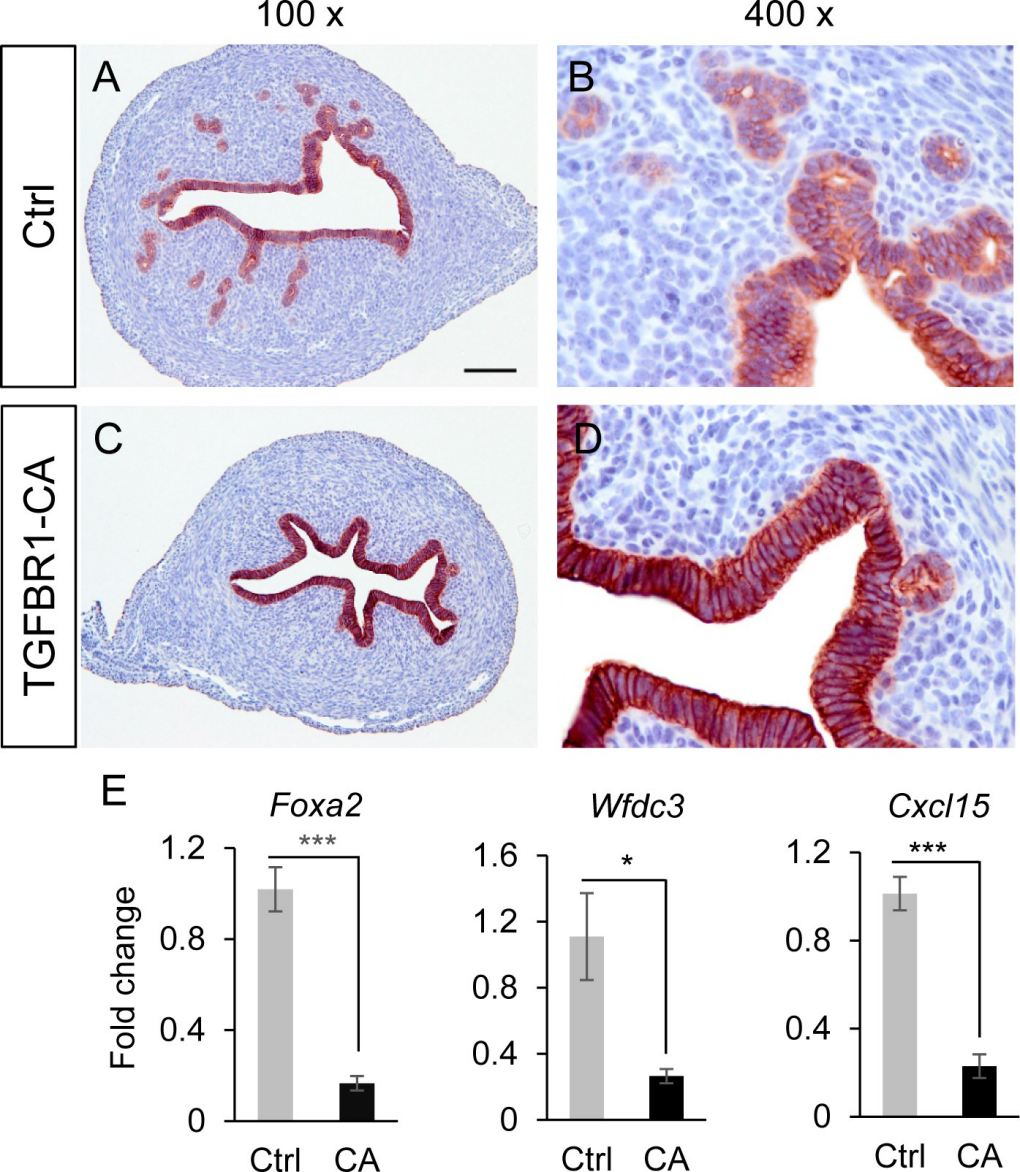
Constitutive activation of TGFBR1 in mouse uterus causes adenogenic defects at D15. (**A**-**D**) Reduced uterine glands in *TGFBR1*^CA flox/flox^; *Pgr*^Cre/+^ mice evidenced by KRT8 staining. Immunohistochemical staining was performed using uterine samples from control (Ctrl; A and B) and *TGFBR1*^CA flox/flox^; *Pgr*^Cre/+^ mice (TGFBR1-CA; C and D) at D15. Panels (B) and (D) represent higher magnification images for panels (A) and (C), respectively. Three mice per group were analyzed and representative images shown. Scale bar is representatively shown in (A) and equals 100 μm (A and C) and 25 μm (B and D). (**E**) Reduced expression of uterine gland-specific genes in the uteri of control (Ctrl) and *TGFBR1*^CA flox/flox^; *Pgr*^Cre/+^ (CA) mice at D15. n = 4–5. *Rpl19* was used as internal control. Data are means ± SEM. *P < 0.05 and ***P < 0.001.

Our earlier study using mice harboring constitutively active TGFBR1 in the uterus (*TGFBR1*^CA flox/flox^; *Pgr*^Cre/+^) has demonstrated several phenotypic changes including disorganized myometrium, enlarged myometrial but reduced stromal compartment and reduced uterine gland formation [13]. This report is to further understand mechanisms contributing to defective uterine gland formation. Since the mouse uterus acquires basic structure by postnatal day 15 (D15), we performed immunostaining of KRT8, an epithelial cell marker, at this timepoint to test the hypothesis that reduction of uterine glands in *TGFBR1*^CA flox/flox^; *Pgr*^Cre/+^ mice was caused by defective adenogenesis. Our results showed a marked reduction of uterine glands in mice with constitutively active TGFBR1 at D15 compared with age-matched controls (Fig 1A–1D). Consistent with the impaired uterine gland formation, expression of mRNA transcripts for uterine gland specific genes including *Foxa2*, *Wfdc3* and *Cxcl15* was significantly decreased in *TGFBR1*^CA flox/flox^; *Pgr*^Cre/+^ uteri compared with controls (Fig 1E).

**Fig 2:**
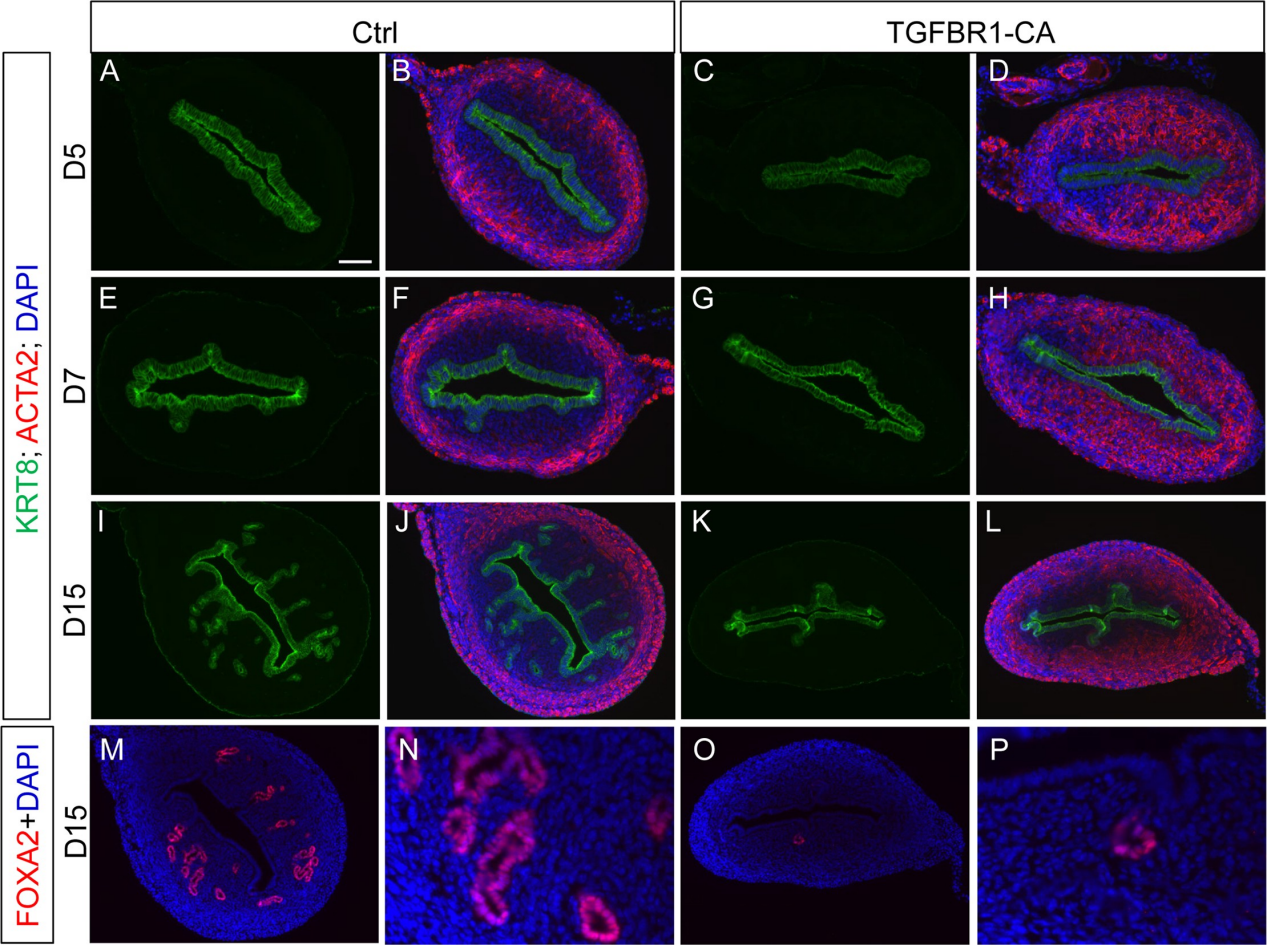
Immunofluorescence microscopy analysis of endometrial glandular and stromal alterations in *TGFBR1*^CA flox/flox^; *Pgr*^Cre/+^ mice during postnatal uterine development. (**A-L**) Immunolocalization of KRT8 (green) and ACTA2 (red) in the uteri of control and *TGFBR1*^CA flox/flox^; *Pgr*^Cre/+^ mice at D5 (A-D), D7 (E-H) and D15 (I-L). (**M-P**) Immunofluorescence of FOXA2 (red) in the uteri of control and *TGFBR1*^CA flox/flox^; *Pgr*^Cre/+^ mice at D15. DAPI (blue) was used to counterstain the nuclei. Three individual samples from each timepoint were examined and representative images depicted. Scale bar is representatively shown in (A) and equals 50 μm (A-H), 100 μm (I-M, and O) and 25 μm (N and P).

To determine how constitutively active TGFBR1 affected early adenogenic event, we performed immunofluorescence microscopy using antibodies directed to KRT8 and ACTA2 at D5, D7 and D15. As expected, uterine glands were not visible at D5 in either control or *TGFBR1*^CA flox/flox^; *Pgr*^Cre/+^ mice (Fig 2A and 2C). Epithelial invagination occurred at D7 in the controls (Fig 2E), whereas this histological change was less evident in the *TGFBR1*^CA flox/flox^; *Pgr*^Cre/+^ uterus (Fig 2G). By D15, abundant uterine glands were detected in the control uterus (Fig 2I). In contrast, uterine glands were sparse in *TGFBR1*^CA flox/flox^; *Pgr*^Cre/+^ mice (Fig 2K), confirming the aforementioned immunohistochemistry finding. FOXA2, a marker specific for glandular epithelia [43] and key regulator of uterine gland development and function [18, 27], was used to verify the identity of uterine glands. Immunofluorescence analysis of FOXA2 revealed the presence of abundant uterine glands in the control but not *TGFBR1*^CA flox/flox^; *Pgr*^Cre/+^ mice (Fig 2M–2P). Further analysis of uterine glands per cross section confirmed that the number of uterine glands in the *TGFBR1*^CA flox/flox^; *Pgr*^Cre/+^ mice was reduced compared with that of controls (0.5 ± 0.0 versus 10.2 ± 3.0; n = 3; *P* < 0.05).

**Fig 3:**
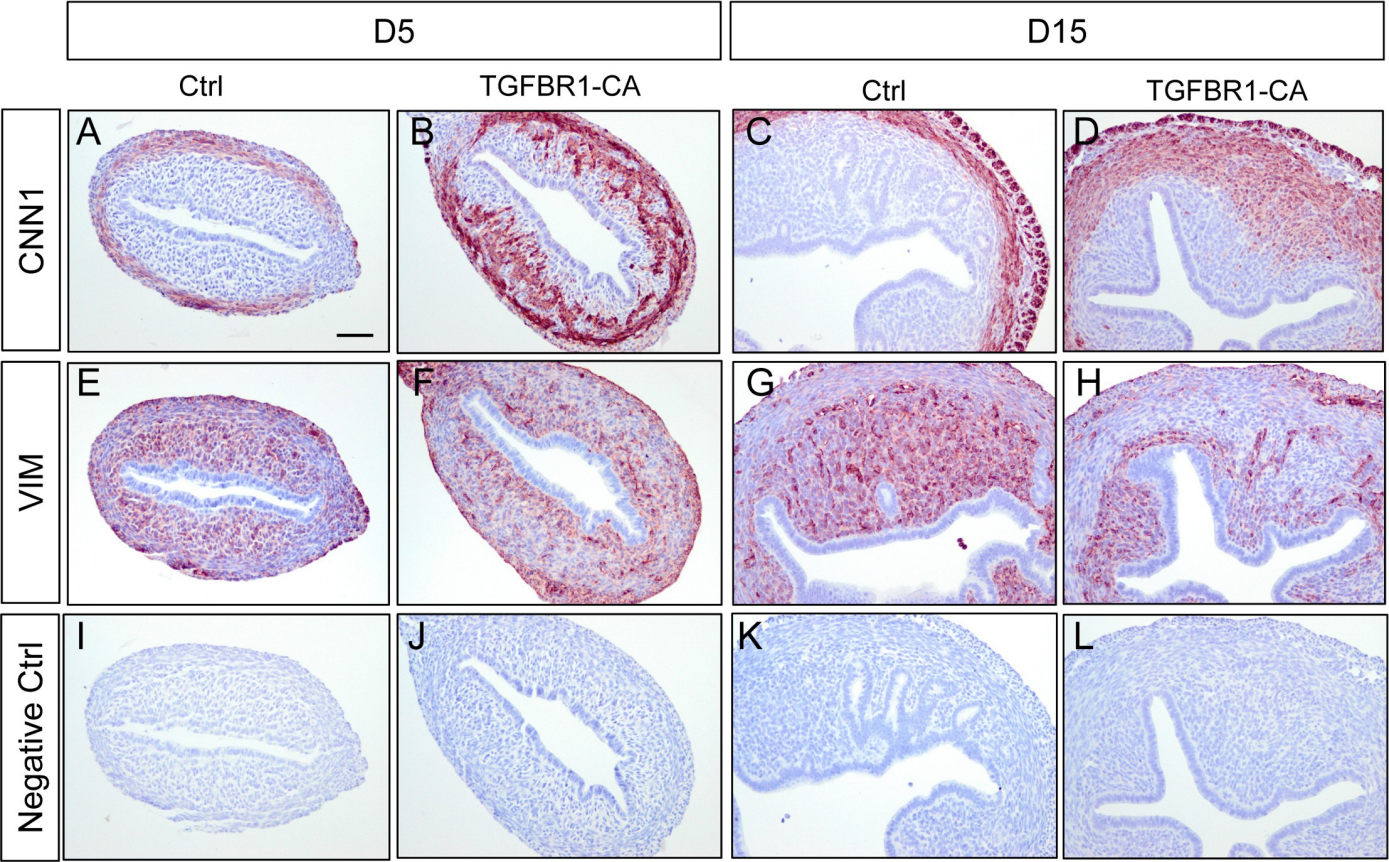
Alteration of early uterine development in *TGFBR1*^CA flox/flox^; *Pgr*^Cre/+^ mice evidenced by CNN1 and VIM localization. (**A**-**H**) Immunohistochemical analysis of CNN1 and VIM in the uteri of control and *TGFBR1*^CA flox/flox^; *Pgr*^Cre/+^ mice at D5 and D15. (**I**-**L**) Negative controls using isotype-matched IgG. Three individual samples from each timepoint were examined. Scale bar is representatively shown in (A) and equals 50 μm (A-L).

Uterine smooth muscle cells differentiate and form myometrial layers after birth in mice. ACTA2 was used to identify myometrial compartment. In the control uterus, well organized myometrial layers and endometrial compartment were visualized by ACTA2 staining from D5 to D15 (Fig 2B, 2F and 2J). However, distinct endometrial and myometrial compartments could not be identified in *TGFBR1*^CA flox/flox^; *Pgr*^Cre/+^ uteri at D5 and D7 using ACTA2 staining (Fig 2D and 2H). Instead, ACTA2-positive cells were in close proximity with luminal epithelial cells in the *TGFBR1*^CA flox/flox^; *Pgr*^Cre/+^ uterus (Fig 2D and 2H), which was in sharp contrast to the control uteri (Fig 2B and 2F). At D15, an endometrial stromal compartment was visible in the *TGFBR1*^CA flox/flox^; *Pgr*^Cre/+^ uterus, surrounded by disoriented ACTA2-positive cells. Disorganized uterine smooth muscle formation in the uteri of *TGFBR1*^CA flox/flox^; *Pgr*^Cre/+^ mice at D5 and D15 was further verified using immunostaining of CNN1 (Fig 3A–3D) and abnormal development of endometrial compartment was evidenced by immunostaining of anti-VIM (Fig 3E–3H). Specific immunoreactive signals were not observed in negative controls using isotype-matched IgG (Fig 3I–3L). To further determine whether activation of TGFB signaling modulated ER and PGR to shape a distinct developmental trajectory, we examined the expression of ER and PGR in the uteri of both control and *TGFBR1*^CA flox/flox^; *Pgr*^Cre/+^ mice at D5 and D15 using immunohistochemistry. Our results showed a similar expression pattern of ER and PGR between control and *TGFBR1*^CA flox/flox^; *Pgr*^Cre/+^ mice (S2 Fig). Collectively, enhanced TGFB signaling in the mouse uterus negatively impacts adenogenesis during early postnatal uterine development, coinciding with altered differentiation of endometrial stromal cells and formation of endometrial compartment.

### Molecular analysis of genes associated with adenogenesis and uterine development in TGFBR1 constitutively active uterus

**Fig 4:**
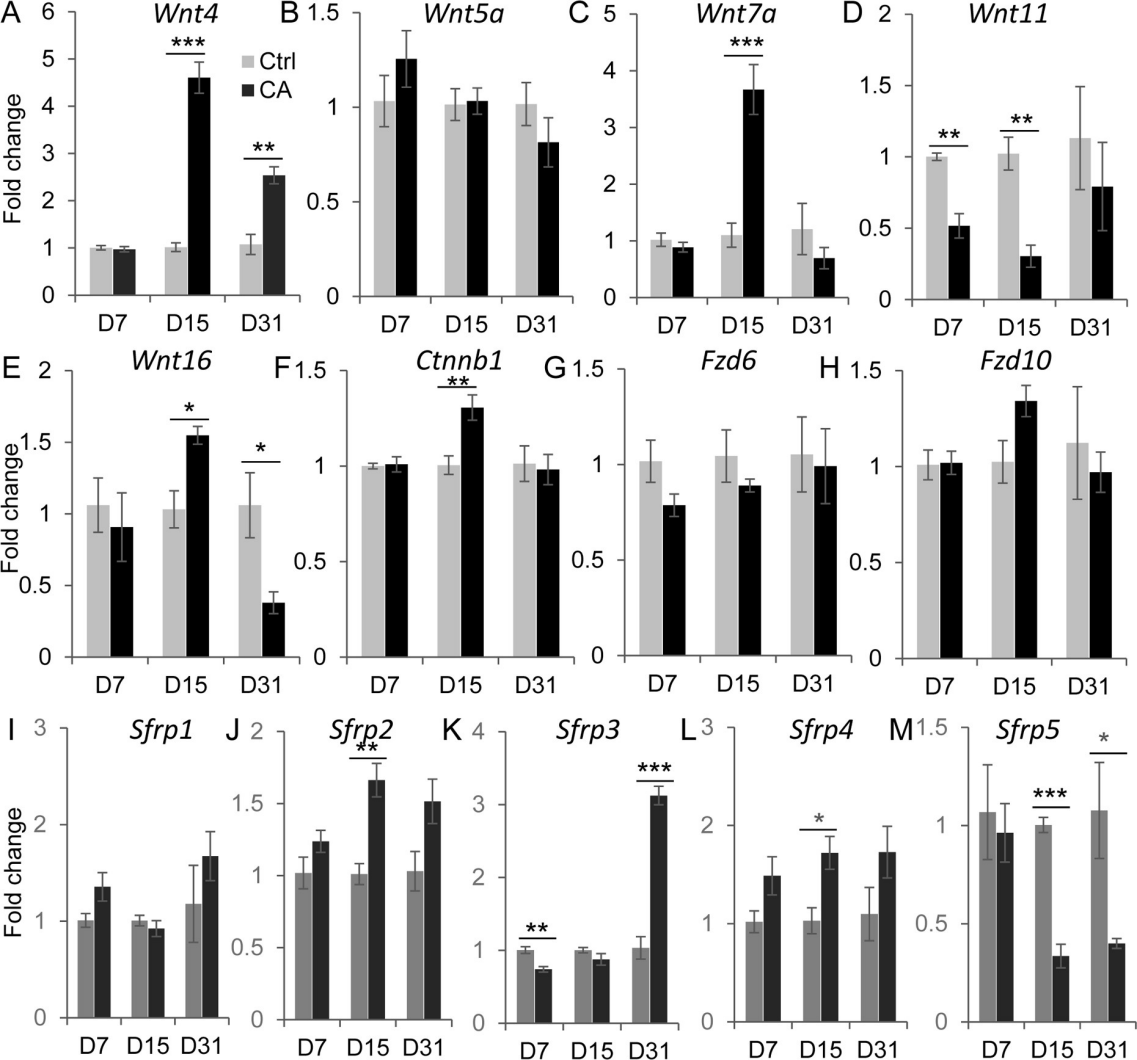
Dysregulation of WNT pathway associated genes in *TGFBR1*^CA flox/flox^; *Pgr*^Cre/+^ uteri. (**A-M**) Transcript levels of *Wnt4*, *Wnt5a*, *Wnt7a*, *Wnt11*, *Wnt16*, *Ctnnb1*, *Fzd6*, *Fzd10* and *Sfrp*1-5 in the uteri of control and *TGFBR1*^CA flox/flox^; *Pgr*^Cre/+^ mice at D7, D15 and D31 were determined by real-time PCR. n = 4–5. *Rpl19* was used as internal control. Data are means ± SEM. *P < 0.05, **P < 0.01 and ***P < 0.001.

To determine the potential mechanism underlying adenogenic defects observed in *TGFBR1*^CA flox/flox^; *Pgr*^Cre/+^ uterus, we compared the transcript levels of genes known to be involved in adenogenesis and uterine development using uterine samples from control and *TGFBR1*^CA flox/flox^; *Pgr*^Cre/+^ mice at D7, D15 and D31. Candidate genes included *Wnt4*, *Wnt5a*, *Wnt7a*, *Wnt11*, *Wnt16*, catenin beta 1 (*Ctnnb1*), frizzled homolog 6 (*Fzd6*), and *Fzd10* [44, 45]. Significant changes in the expression of the above genes were not found in *TGFBR1*^CA flox/flox^; *Pgr*^Cre/+^ uteri at D7 except *Wnt11* (Fig 4D). At D15, mRNA levels of *Wnt4* (Fig 4A), *Wnt7a* (Fig 4C) and *Ctnnb1* (Fig 4F) were increased, whereas expression of *Wnt11* (Fig 4D) was reduced, in *TGFBR1*^CA flox/flox^; *Pgr*^Cre/+^ uteri compared with age-matched controls. At D31, a significant difference was only detected in *Wnt4* (Fig 4A) and *Wnt16* (Fig 4E) mRNA expression between control and *TGFBR1*^CA flox/flox^; *Pgr*^Cre/+^ uteri. As secreted Frizzled related proteins (SFRPs) can bind to WNT or Frizzled membrane receptors to negatively modulate WNT signaling activity [46], we determined the mRNA expression of *Sfrp1-5* in control and *TGFBR1*^CA flox/flox^; *Pgr*^Cre/+^ uteri at D7, D15 and D31. Results are shown in Fig 4I–4M. Differential changes of the *Sfrp* genes in the uteri were observed between control and *TGFBR1*^CA flox/flox^; *Pgr*^Cre/+^ mice among different developmental stages. The results revealed complex alterations of *Sfrp* expression in the uteri of *TGFBR1*^CA flox/flox^; *Pgr*^Cre/+^ mice versus controls. These findings suggest a potential link between the dysregulation of adenogenic gene expression and the defective adenogenesis in *TGFBR1*^CA flox/flox^; *Pgr*^Cre/+^ mice.

**Fig 5:**
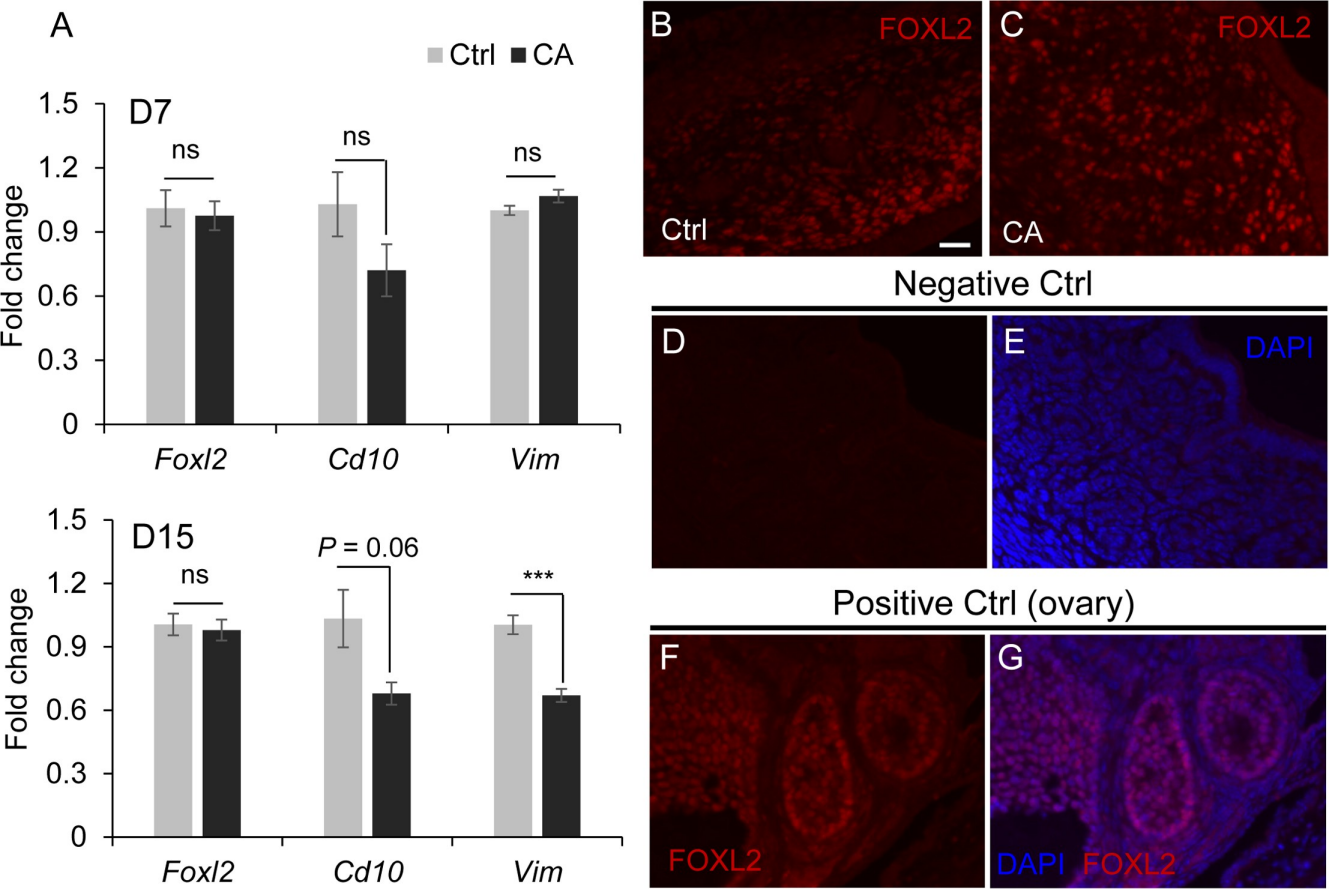
Expression of FOXL2 is not altered in *TGFBR1*^CA flox/flox^; *Pgr*^Cre/+^ uterus. (**A**) Real-time PCR analysis of *Foxl2*, *Cd10* and *Vim* in the uteri of control and *TGFBR1*^CA flox/flox^; *Pgr*^Cre/+^ mice at D7 and D15. n = 4–5. *Rpl19* was used as internal control. Data are means ± SEM, Ns, not significant. ***P < 0.001. (**B** and **C**) Immunofluorescence of FOXL2 in control and *TGFBR1*^CA flox/flox^; *Pgr*^Cre/+^ uteri. (**D** and **E**) Negative controls where FOXL2 antibody was replaced by isotype-matched IgG. (**F** and **G**) Positive controls using wild type ovaries. Scale bar is representatively shown in (B) and equals 20 μm (B-G).

*Foxl2*, a critical gene in ovarian development and function [47], has been reported to be expressed in the uterus and required for uterine maturation [48]. Because conditional deletion of *Foxl2* using *Pgr*-Cre also shows a similarly enlarged ACTA2-positive component [13, 48], we sought to determine whether the defective cellular differentiation in the *TGFBR1*^CA flox/flox^; *Pgr*^Cre/+^ uterus was linked to altered FOXL2 expression. Our real-time PCR assay showed comparable uterine mRNA levels of *Foxl2* between control and *TGFBR1*^CA flox/flox^; *Pgr*^Cre/+^ mice at both D7 and D15, despite the reduction (or trended reduction) of expression of stromal cell markers [49], cluster of differentiation 10 (*Cd10*) and vimentin (*Vim*) at D15 (Fig 5A). Immunofluorescence showed that FOXL2 was mainly expressed in uterine stromal cells in control mice (Fig 5B). In the *TGFBR1*^CA flox/flox^; *Pgr*^Cre/+^ mice, comparable FOXL2 signals were detected in the stromal compartment (Fig 5C). Negative controls where FOXL2 antibody was replaced by isotype-matched IgG are depicted in Fig 5D and 5E. Positive controls using wild type ovaries showed specific staining of FOXL2 in ovarian granulosa cells (Fig 5F and 5G). This result indicates that overactivation of TGFBR1 may function independently of FOXL2 or TGFBR1 functions downstream of FOXL2 in the mouse uterus.

### Luminal epithelial cell proliferation is increased in TGFBR1 constitutively active uterus

**Fig 6:**
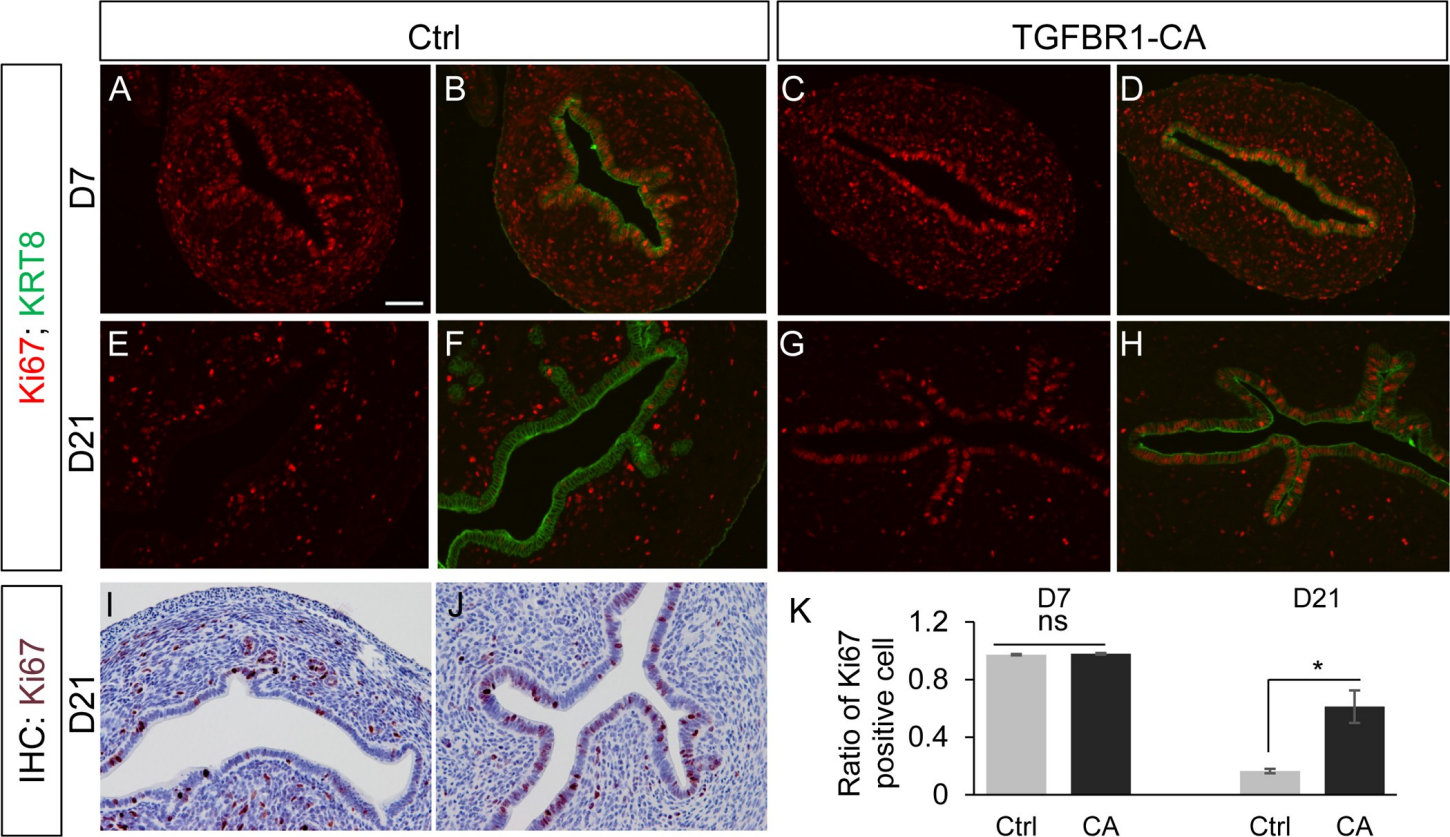
Analysis of uterine luminal epithelial cell proliferation in control and *TGFBR1*^CA flox/flox^; *Pgr*^Cre/+^ uteri. (**A-H**) Immunofluorescence of Ki67 (red) and KRT8 (green) in control and *TGFBR1*^CA flox/flox^; *Pgr*^Cre/+^ mice at D7 (A-D) and D21 (E-H). (**I** and **J**) Representative immunohistochemical staining of Ki67 in the control and *TGFBR1*^CA flox/flox^; *Pgr*^Cre/+^ uteri at D21, respectively. The sections were counterstained with hematoxylin. Scale bar is representatively shown in (A) and equals 50 μm (A-J). (**K**) Quantification of Ki67-positive cells in the luminal epithelia of control and *TGFBR1*^CA flox/flox^; *Pgr*^Cre/+^ uteri at D7 and D21. n = 3 per group. Data represent the ratio of the number of Ki67-positive cells to the total number of cells counted. Data are mean ± SEM. *P < 0.05 versus corresponding controls. Ns, not significant.

Epithelial proliferation may play a permissive role in uterine adenogenesis [29, 50]. Thus, impaired luminal epithelial cell proliferation could potentially lead to the observed adenogenic defects. To determine whether luminal epithelial cell proliferation was altered in *TGFBR1*^CA flox/flox^; *Pgr*^Cre/+^ uteri, we examined the proliferative status of uterine luminal epithelial cells at D7 and D21 in control and *TGFBR1*^CA flox/flox^; *Pgr*^Cre/+^ mice using immunostaining of Ki67, a cell proliferation marker. Our results showed that uterine luminal epithelial cells were highly proliferative at D7 in both control and *TGFBR1*^CA flox/flox^; *Pgr*^Cre/+^ uteri (Fig 6A–6D). The proliferation of uterine epithelial cells in D21 control mice was low (Fig 6E, 6F and 6I), consistent with a previous report [29]. However, abundant Ki67-positive cells were present in the luminal epithelia of *TGFBR1*^CA flox/flox^; *Pgr*^Cre/+^ uteri (Fig 6G, 6H and 6J). Further quantitative analysis did not reveal a difference in the number of Ki67-positive luminal epithelial cells between control and *TGFBR1*^CA flox/flox^; *Pgr*^Cre/+^ mice at D7, but showed that the number of Ki67-positive luminal epithelial cells was increased in *TGFBR1*^CA flox/flox^; *Pgr*^Cre/+^ uteri versus controls at D21 (Fig 6K). Therefore, sustained activation of TGFBR1 in the mouse uterus does not impede uterine luminal epithelial cell proliferation.

### Fibrotic changes in the TGFBR1 constitutively active uterus

**Fig 7:**
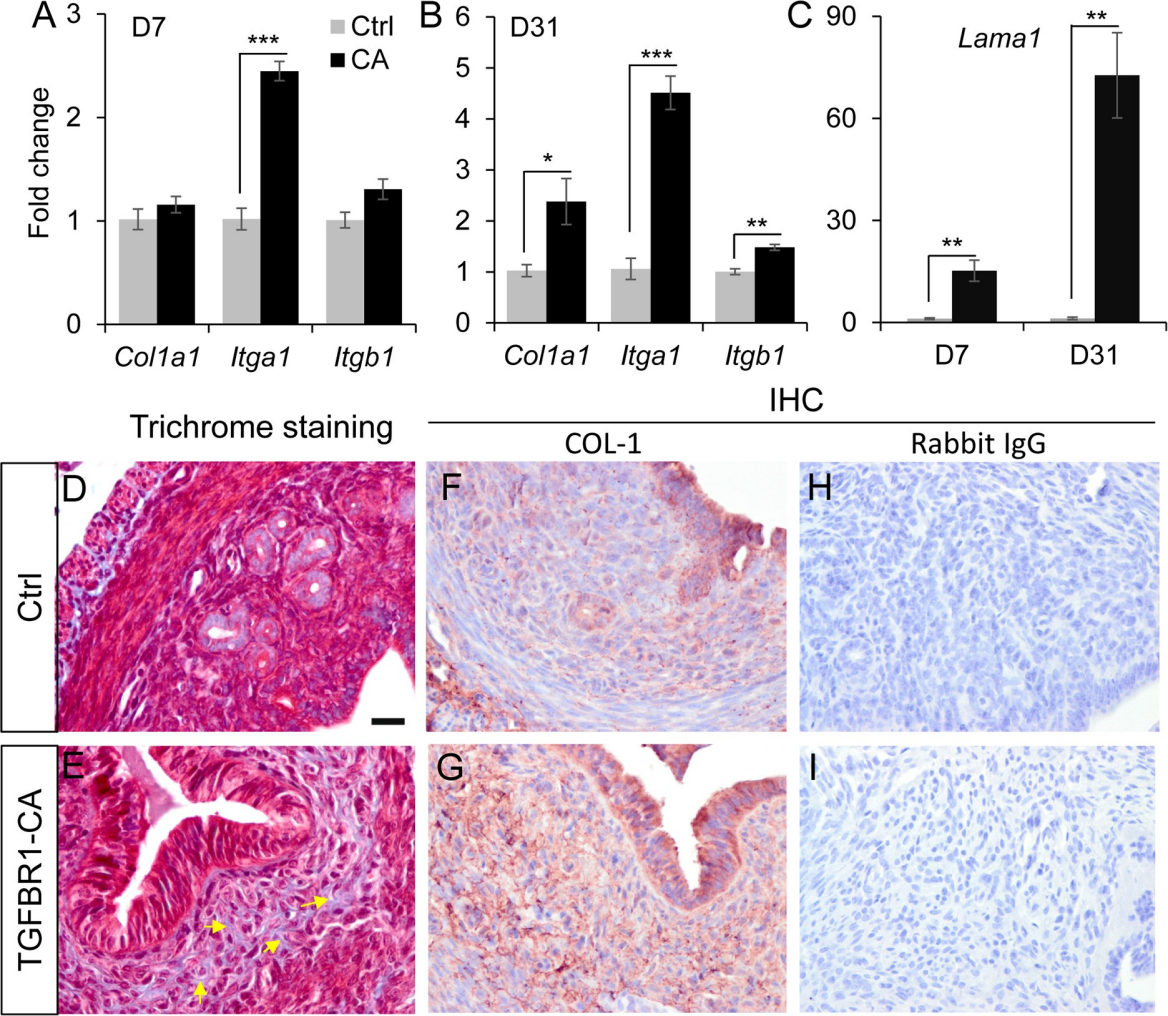
Increased expression of extracellular matrix related genes and fibrotic changes in *TGFBR1*^CA flox/flox^; *Pgr*^Cre/+^ uteri. (**A**) *Itga1* mRNA levels were elevated in *TGFBR1*^CA flox/flox^; *Pgr*^Cre/+^ uteri at D7. (**B**) *Col1a1*, *Itga1* and *Itgb1* mRNA abundance was increased in *TGFBR1*^CA flox/flox^; *Pgr*^Cre/+^ uteri at D31. (**C**) *Lama1* mRNA levels were increased in *TGFBR1*^CA flox/flox^; *Pgr*^Cre/+^ uteri. n = 4 per group. Data are means ± SEM. *P < 0.05, **P < 0.01 and ***P < 0.001. (**D** and **E**) Trichrome staining of uteri from control and *TGFBR1*^CA flox/flox^; *Pgr*^Cre/+^ mice at 1 month of age. Note increased blue staining in uterine stroma of *TGFBR1*^CA flox/flox^; *Pgr*^Cre/+^ mice versus controls. (**F**-**I**) Immunohistochemistry of COL-1 in the uteri from control and *TGFBR1*^CA flox/flox^; *Pgr*^Cre/+^ mice at 1 month of age. Negative controls using rabbit IgG are shown in (H) and (I). Three individual samples from each group were examined. Scale bar is representatively shown in (D) and equals 20 μm (D-I).

**Fig 8:**
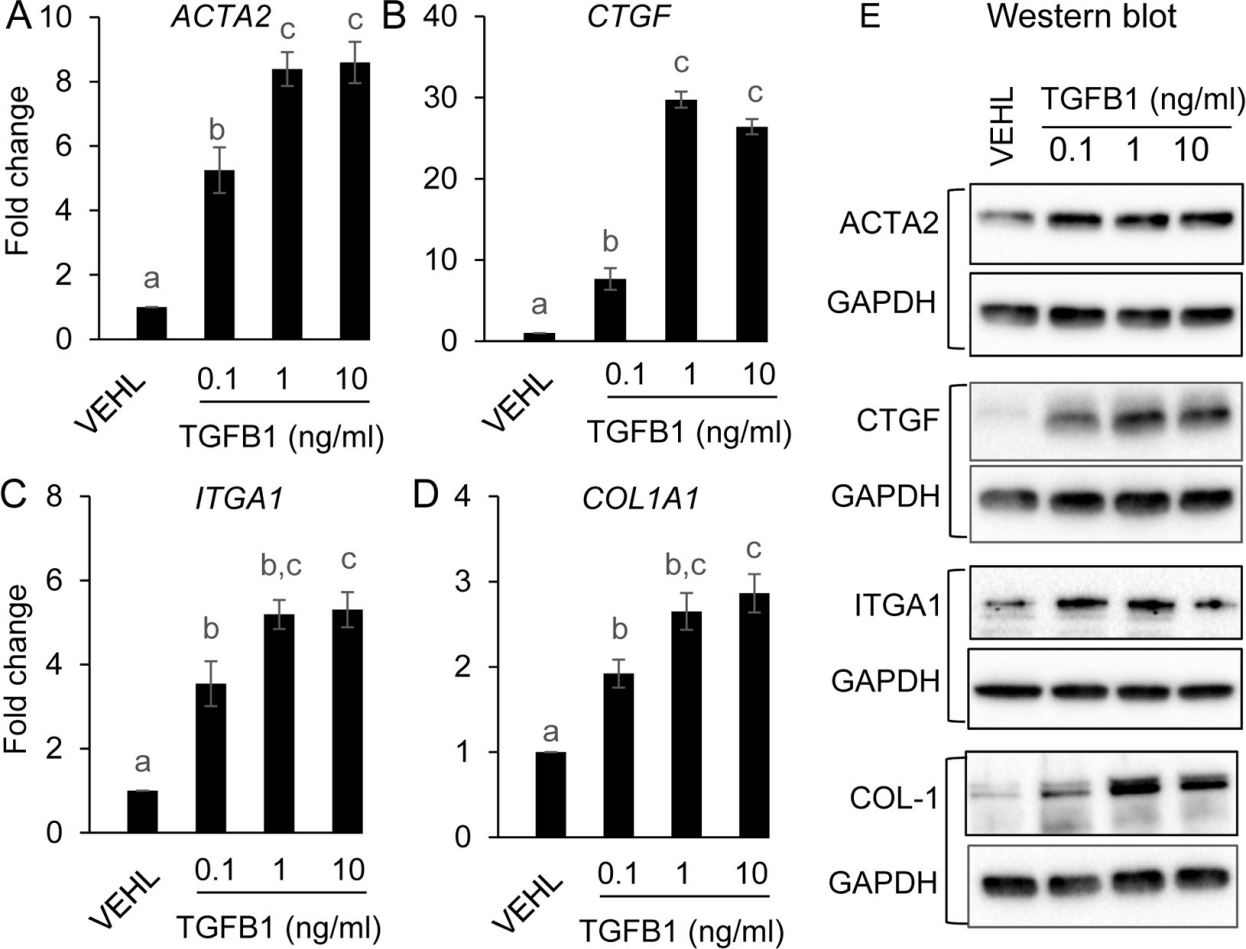
TGFB1 induces the expression of fibrotic genes in human endometrial stromal cells. (**A-D**) Altered mRNA expression of *ACTA2* (A), *CTGF* (B), *ITGA1* (C) and *COL1A1* (D) in human endometrial stromal cells upon TGFB1 treatment. Human endometrial stromal cells (T-HESC) were cultured overnight, serum starved, and treated with TGFB1 (0.1–10 ng/ml). Cells were collected after 24 h of treatment and subject to real-time PCR analysis. Three independent culture experiments were performed. *RPL19* was used as internal control. Data are means ± SEM. Bars without the same superscripts are significantly different (*P* < 0.05). (**E**) Western blot analysis of the effect of TGFB1 treatment on protein expression of ACTA2, CTGF, ITGA1 and COL-1 in human endometrial stromal cells. At least three independent cell culture experiments were performed. Uncropped images are shown in S3 Fig.

Consistent with the well-established role of TGFB ligands as fibrotic proteins [51], transcript levels for genes encoding collagen and laminin (*Col1a1* and *Lama1*) were significantly increased in *TGFBR1*^CA flox/flox^; *Pgr*^Cre/+^ uteri compared with controls at D7 and/or D31 (Fig 7A–7C). Integrins link extracellular matrix (ECM) to cytoskeleton. Here we demonstrated that the mRNA expression of *Itga1* and *Itgb1* was increased in the uteri with overactivation of TGFBR1 (Fig 7A and 7B). To visualize collagen-containing fibers in the uterus, we performed Trichrome staining using uteri from both control and *TGFBR1*^CA flox/flox^; *Pgr*^Cre/+^ mice at 1 month of age. Results showed increased blue collagen fibers in the stromal compartment of *TGFBR1*^CA flox/flox^; *Pgr*^Cre/+^ mice compared with controls (Fig 7D and 7E). Immunohistochemistry using anti-COL-1 antibody also revealed increased collagen protein expression in *TGFBR1*^CA flox/flox^; *Pgr*^Cre/+^ uteri versus controls (Fig 7F and 7G). Negative controls using rabbit IgG are shown in Fig 7H and 7I. These data suggest that enhanced TGFB signaling leads to fibrotic changes in mouse endometrium. To independently test the role of TGFB signaling in the regulation of the fibrotic genes, we used human uterine stromal cells and showed that TGFB1 (0.1–10 ng/ml) treatment significantly increased the mRNA levels of *ACTA2*, *CTGF* [52], *ITGA1* and *COL1A1* after 24 h of treatment (Fig 8A–8D). Western blot analysis confirmed the stimulatory effect of TGFB1 on the protein expression of ACTA2, CTGF, ITGA1 and COL-1 (Fig 8E and S3 Fig). These results suggest that chronic activation of TGFBR1 leads to fibrotic changes in endometrial stromal compartment, which impairs endometrial cell function.

### Mosaic activation of TGFBR1 in the mouse uterus alleviates adenogenic defects

**Fig 9:**
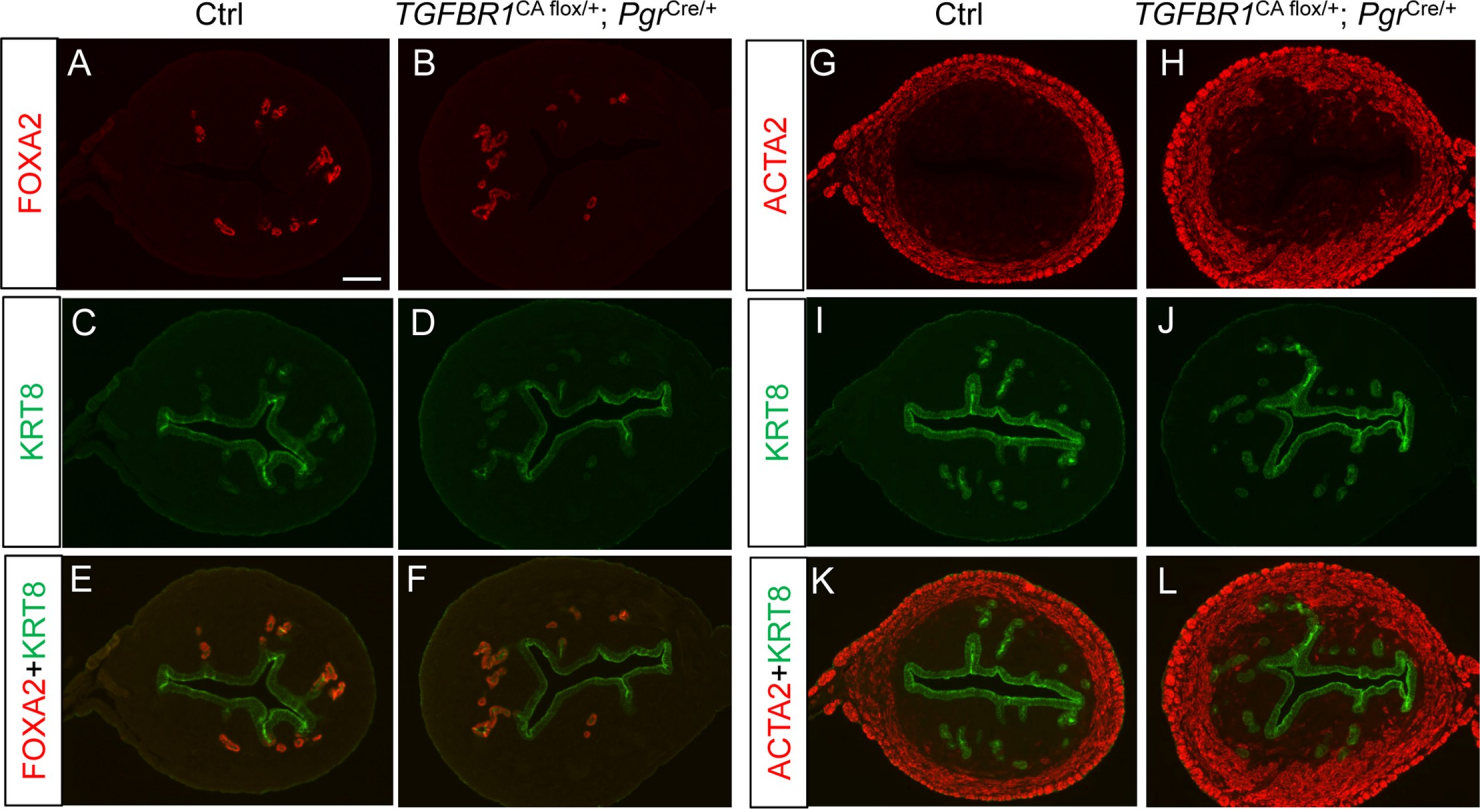
Uterine adenogenesis in *TGFBR1*^CA flox/+^; *Pgr*^Cre/+^ mice. (**A-F**) Immunofluorescence detection of FOXA2 (red) and KRT8 (green) in the uteri of control and *TGFBR1*^CA flox/+^; *Pgr*^Cre/+^ mice. Note that uterine adenogenesis was detectable in *TGFBR1*^CA flox/+^; *Pgr*^Cre/+^ mice at D15, with glandular epithelia marked by FOXA2. (**G-L**) Immunofluorescence detection of ACTA2 (red) and KRT8 (green) in the uteri of control and *TGFBR1*^CA flox/+^; *Pgr*^Cre/+^ mice. Scale bar is representatively shown in (A) and equals 100 μm.

The aforementioned uterine fibrotic changes and the unimpeded proliferation of epithelial cells suggest that the adenogenic defects observed in *TGFBR1*^CA flox/flox^; *Pgr*^Cre/+^ mice are caused by altered uterine stromal cell differentiation and formation of endometrial stromal cell compartment. We anticipated that a reduction of the number of endometrial stromal cells expressing the *TGFBR1*^*CA*^ transgene would alleviate the adenogenic defects. Therefore, we generated a mouse model with activation of TGFBR1 in presumably half of the complement of uterine cells (i.e., *TGFBR1*^CA flox/+^; *Pgr*^Cre/+^) due to the targeting of *TGFBR1*^CA^ to the X-linked *Hprt* locus and X-chromosome inactivation in the females during development [33, 53]. Expression of TGFBR1 mRNA levels in the uteri from *TGFBR1*^CA flox/+^; *Pgr*^Cre/+^ mice at the age of 1 month were analyzed using real-time PCR, demonstrating the expression of the transgene (S4 Fig). As expected, adenogenesis occurred in *TGFBR1*^CA flox/+^; *Pgr*^Cre/+^ mice, with readily detectable uterine glands at D15 by immunofluorescence using antibodies against both KRT8 and FOXA2 (Fig 9B, 9D and 9F). Double immunofluorescence of ACTA2 and KRT8 showed the formation of uterine glands and enlarged muscle component in *TGFBR1*^CA flox/+^; *Pgr*^Cre/+^ mice (Fig 9H, 9J and 9L). Age-matched *TGFBR1*^CA flox/+^ mice were included as controls (Fig 9A, 9C, 9E, 9G, 9I and 9K).

## Discussion

Toward the goal to defining the role of TGFB signaling in uterine function, we created a mouse model that harbors a constitutively active TGFBR1 in the uterus using *Pgr*-Cre recombinase in a previous report [13]. In addition to myometrial abnormality, uterine gland development was impaired in these mice. This study was to follow up the adenogenic defects in these mice and identify the underpinning cellular and molecular basis.

The uterus differentiates from Müllerian duct. In general, Müllerian duct differentiation is a highly coordinated event regulated by genes including, but not limited to, LIM homeobox protein 1 (*Lim1*), paired box 2 (*Pax2*), *Wnt9b*, *Wnt5a* and homeobox (*Hox*) genes [22, 54–57]. Adenogenesis, a physiologic process of uterine gland formation, occurs postnatally in mice [50]. Recent studies from the Behringer laboratory demonstrated that uterine gland formation is a continuous process in mice [58]. The uterus contains simple epithelium and supporting mesenchyme, with no endometrial glands at birth. Adenogenesis occurs via invaginations of luminal epithelium by D6. Uterine glands are evident on D7, and the uterus acquires essential structures (i.e., myometrium and endometrium consisting of stroma and glands) by D15 [59, 60]. Therefore, we examined adenogenesis in *TGFBR1*^CA flox/flox^; *Pgr*^Cre/+^ mice during the critical period of uterine gland formation and revealed abnormal uterine stromal cell differentiation and formation of uterine stromal compartment. The altered uterine cell differentiation suggests that uterine cells are sensitive to TGFBR1 overactivation. However, a potential contribution of altered uterine epithelial-mesenchymal interaction to the abnormal ACTA2 expression could not be excluded.

It has been reported that WNT pathway components including WNTs and CTNNB1 are crucial for uterine gland development [22, 23, 61, 62]. For examples, both WNT5A and WNT7A are required for uterine gland formation and WNT7A is also essential for normal patterning of the uterus during development [22, 23, 25]. WNT4, which is abundantly expressed in the stroma of neonatal mouse uterus [44], has also been implicated in uterine gland formation. A recent study suggests that DICER1 may be a regulator of uterine gland development, based on the observation that conditional knockout of *Dicer1* using *Pgr*-Cre leads to loss of glandular epithelium in the uterus [63]. However, how loss of DICER1 contributes to this phenotype requires further investigation. TGFB signaling interacts with WNTs [64]. Therefore, we examined the expression of WNT pathway components in the *TGFBR1*^CA flox/flox^; *Pgr*^Cre/+^ uterus. Interestingly, despite the altered cell properties, the expression of *Ctnnb1* and a number of adenogenesis associated genes in the uteri of *TGFBR1*^CA flox/flox^; *Pgr*^Cre/+^ mice was comparable to that of controls at D7, except a reduction of *Wnt11* and *Sfrp3* mRNA levels. Although *Wnt11* is expressed in uterine epithelium, conditional ablation of *Wnt11* does not impair uterine gland genesis [44], suggesting the adenogenic program may be functional at the beginning of adenogenesis. It was noteworthy that several *Wnt* and *Sfrp* genes were dysregulated at D15 and/or D31. Although the specific role of WNT11 in uterine development is not clear, its reduction in the *TGFBR1*^CA flox/flox^; *Pgr*^Cre/+^ uteri could reflect the decreased epithelial components in these mice. It is intriguing, however, *Wnt4*, *Wnt7a*, *Wnt16* and *Ctnnb1* mRNA levels were increased in the *TGFBR1*^CA flox/flox^; *Pgr*^Cre/+^ uteri at D15. Since these genes are essential for normal uterine gland development [21–23, 25, 44, 61], it is tempting to speculate that their upregulation in the *TGFBR1*^CA flox/flox^; *Pgr*^Cre/+^ uteri may represent a compensatory mechanism.

Elegant work has shown that progesterone administration in a limited time window during postnatal uterine development results in depletion of endometrial glands, accompanied by inhibition of luminal epithelial cell proliferation [17, 29]. To determine whether the impaired adenogensis in our model is potentially caused by compromised luminal epithelial cell proliferation, we assessed the proliferation status of uterine epithelial cells and demonstrated that the proliferation of luminal epithelial cells was not impeded at D7. In fact, increased number of Ki67-posittive luminal epithelial cells was found in the *TGFBR1*^CA flox/flox^; *Pgr*^Cre/+^ uterus at D21. Thus, this finding further indicates that the adenogenic program may remain functional in the *TGFBR1*^CA flox/flox^; *Pgr*^Cre/+^ uterus at the beginning of uterine gland formation. However, dysregulation of *Wnt* pathway-related genes at D15 indicates that this program may be compromised with the progression of adenogenic process.

Uterine epithelial-mesenchymal interaction is critically important for uterine development and the specification and maintenance of the integrity of mesenchymal compartments [22, 65]. An interesting finding of this study was that uterine stromal cell differentiation was altered at an early developmental stage (i.e., D5) in the *TGFBR1*^CA flox/flox^; *Pgr*^Cre/+^ uteri, characterized by unrecognizable endometrial stromal compartment at D5 and D7. Consequently, ACTA2-positive cells were in close proximity with luminal epithelia in the *TGFBR1*^CA flox/flox^; *Pgr*^Cre/+^ mice, as is potentially detrimental to the formation of uterine glands. Although an endometrial stromal compartment was morphologically identifiable at D15, it was restricted and comprised sparse uterine glands. It has been well established that TGFB signaling is a potent driver of fibrotic responses in a variety of organs/tissues [51, 66, 67]. Our human endometrial cell culture experiment extends the findings from mice by revealing the regulation of several pro-fibrogenic genes by TGFB signaling. We found that TGFB1 induces the expression of *ACTA2*, *COL1A1*, *ITGA1* and *CTGF*, in line with previous reports that TGFB/SMAD signaling promotes fibrosis by inducing transcription of pro-fibrogenic genes [13, 68, 69]. Of note, CTGF is an essential mediator of tissue remodeling and fibrosis [52]. Upregulation of *CTGF* by TGFB in human endometrial cells is consistent with our previous report that *Ctgf* transcripts are increased in *TGFBR1*^CA flox/flox^; *Pgr*^Cre/+^ uteri [13]. It is thus plausible that the changes of the expression of genes encoding matrix proteins, integrins and smooth-muscle filament proteins in the *TGFBR1*^CA flox/flox^; *Pgr*^Cre/+^ uteri may influence the matrix properties and promote the development of a smooth muscle-like barrier that is less permissive to uterine gland branching/formation compared to a normal uterine microenvironment where the “soil” for adenogenesis consists primarily of normal stromal cells. Furthermore, by taking advantage of a mouse model where *TGFBR1*^CA^ transgene was expressed in presumably half of the complement of uterine cells, we found that adenogenesis occurred in these mice. Results from this model provided circumstantial evidence that impaired adenogenesis is caused by altered endometrial stroma property. This evidence supports the notion that abnormal mesenchymal cell differentiation caused by overactivation of TGFBR1 negatively impacts uterine microenvironment and adenogenesis. The model may serve as a tool to study the effect of activation of TGFBR1 in partial versus full complement of uterine cells. However, a potential caveat for this model is that reduction of cells expressing *TGFBR1*^CA^ may occur not only in the stromal compartment but also in the epithelial lineage due to the known expression pattern of *Pgr*-Cre in the uterus. This possibility needs to be further investigated. In addition, although the defects in early postnatal adenogenesis observed in mice harboring constitutively active TGFBR1 signify the importance of appropriately controlled TGFB signaling activity in uterine development, findings from this mouse model should not be interpreted as the physiologic role of TGFB signaling in adenogenesis.

In summary, our findings suggest that altered differentiation of uterine stromal cells and formation of endometrial stromal compartment resulting from sustained activation of TGFBR1 is a key contributing factor to the adenogenic defects in these mice. This study has potential implication in understanding the pathological role of TGFB signaling in uterine disease associated with endometrial dysfunction.

## Supporting information

S1 Fig Induction of *IGFBP1* mRNA expression in THESC upon treatment with 8-bromoadenosine 3', 5'-cyclic monophosphate (8-Br-cAMP). THESCs were treated with vehicle (VEHL) or 8-Br-cAMP (0.5 mM) for 6 days. Four independent cell culture experiments were performed. Data are means ± SEM. **P < 0.01.
(TIF)

S2 Fig Expression of ER and PGR in the uteri of mice with constitutively active TGFBR1 during early uterine development. (**A**-**H**) Immunohistochemical analysis of ER and PGR in the uteri of control and *TGFBR1*^CA flox/flox^; *Pgr*^Cre/+^ mice at D5 and D15. Three individual samples from each timepoint were examined. Scale bar is representatively shown in (A) and equals 50 μm (A-H).
(TIF)

S3 Fig Uncropped western blot images. Full images for western blot shown in Fig 8E. First row shows the blots for ACTA2, CTGF, ITGA1 and COL-1 proteins, and the second row shows the corresponding GAPDH. Dashed boxes indicate target bands with expected molecular weights.
(TIF)

S4 Fig Expression of *TGFBR1*^CA^ in the uteri of *TGFBR1*^CA flox/+^; *Pgr*^Cre/+^ mice at the age of 1 month. n = 4 for control and n = 6 for *TGFBR1*^CA flox/+^; *Pgr*^Cre/+^ mice. *Rpl19* was used as internal control. Data are means ± SEM. ***P < 0.001.
(TIF)
