# Supplementary figures and images for: Glandular defects in the mouse uterus with sustained activation of TGF-beta signaling is associated with altered differentiation of endometrial stromal cells and formation of stromal compartment
Source: PLoS One. 2018 Dec 14;13(12):e0209417. doi: 10.1371/journal.pone.0209417 (PMC6294433; doi:10.1371/journal.pone.0209417)

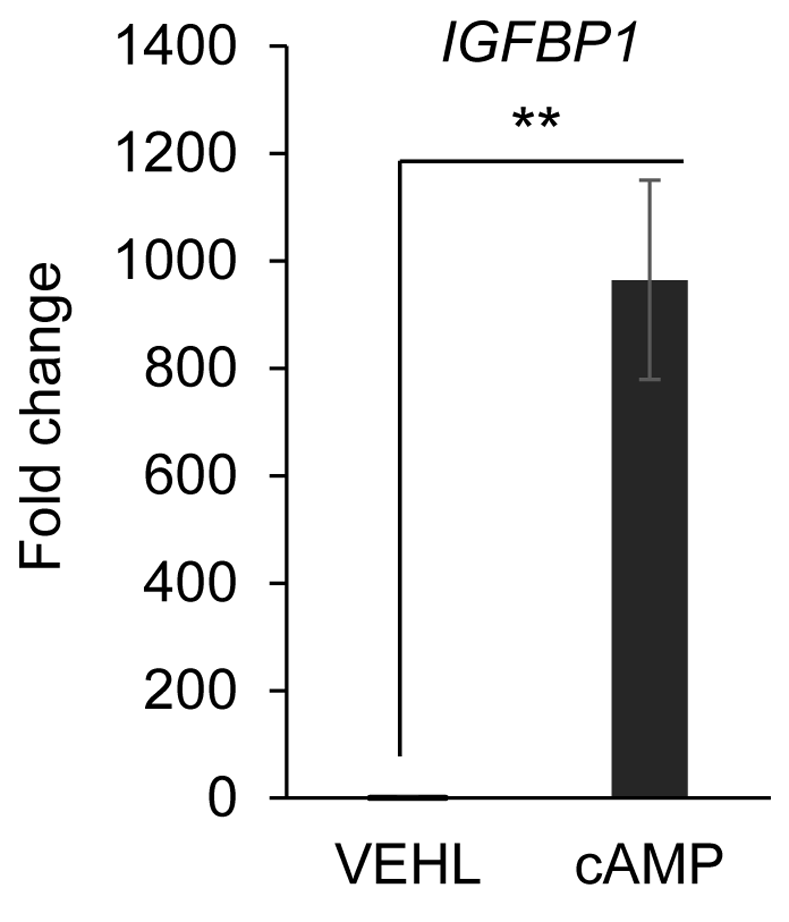

Supplement: S1 Fig — THESCs were treated with vehicle (VEHL) or 8-Br-cAMP (0.5 mM) for 6 days. Four independent cell culture experiments were performed. Data are means ± SEM. **P < 0.01. (TIF) [file pone.0209417.s001.tif]

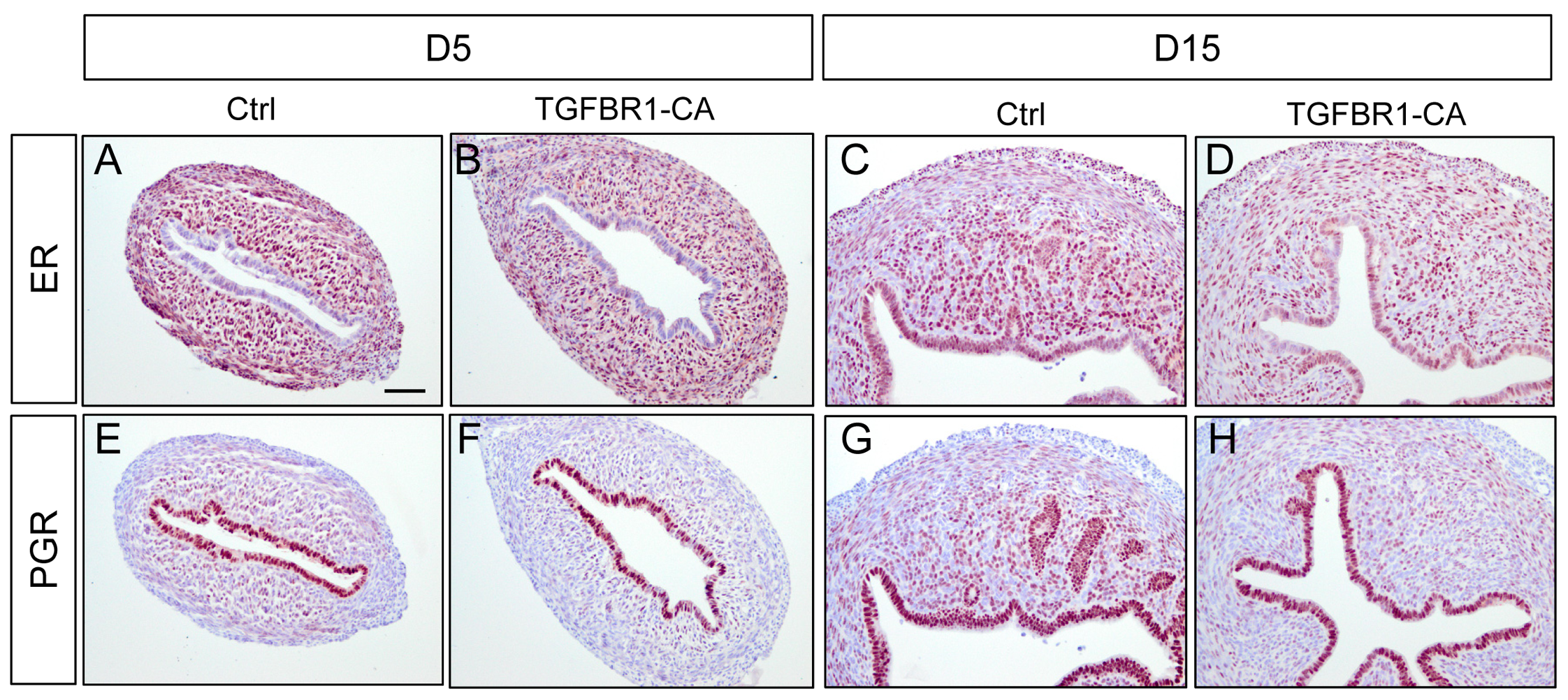

Supplement: S2 Fig — (A-H) Immunohistochemical analysis of ER and PGR in the uteri of control and TGFBR1CA flox/flox; PgrCre/+ mice at D5 and D15. Three individual samples from each timepoint were examined. Scale bar is representatively shown in (A) and equals 50 μm (A-H). (TIF) [file pone.0209417.s002.tif]

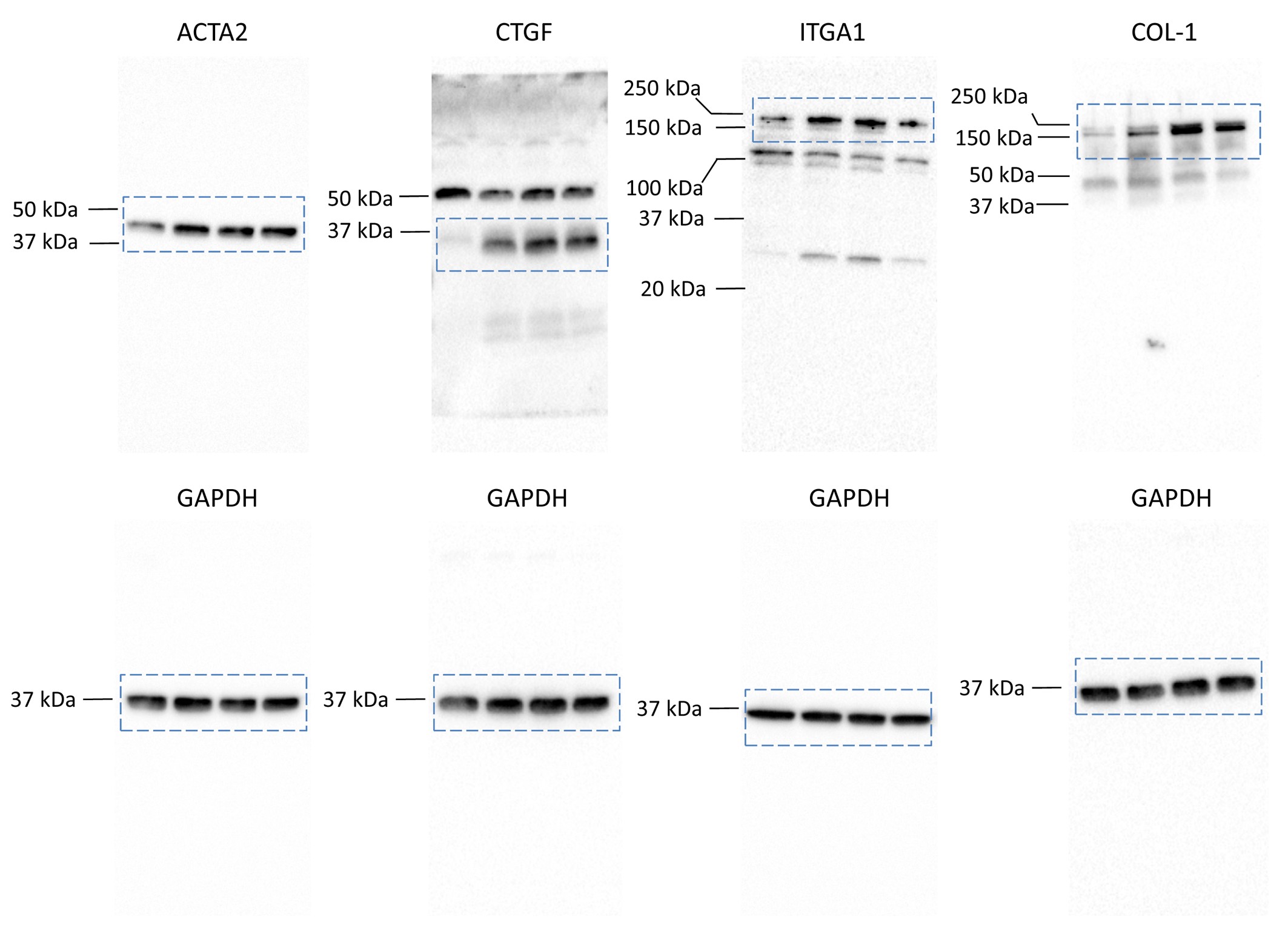

Supplement: S3 Fig — Full images for western blot shown in Fig 8E. First row shows the blots for ACTA2, CTGF, ITGA1 and COL-1 proteins, and the second row shows the corresponding GAPDH. Dashed boxes indicate target bands with expected molecular weights. (TIF) [file pone.0209417.s003.tif]

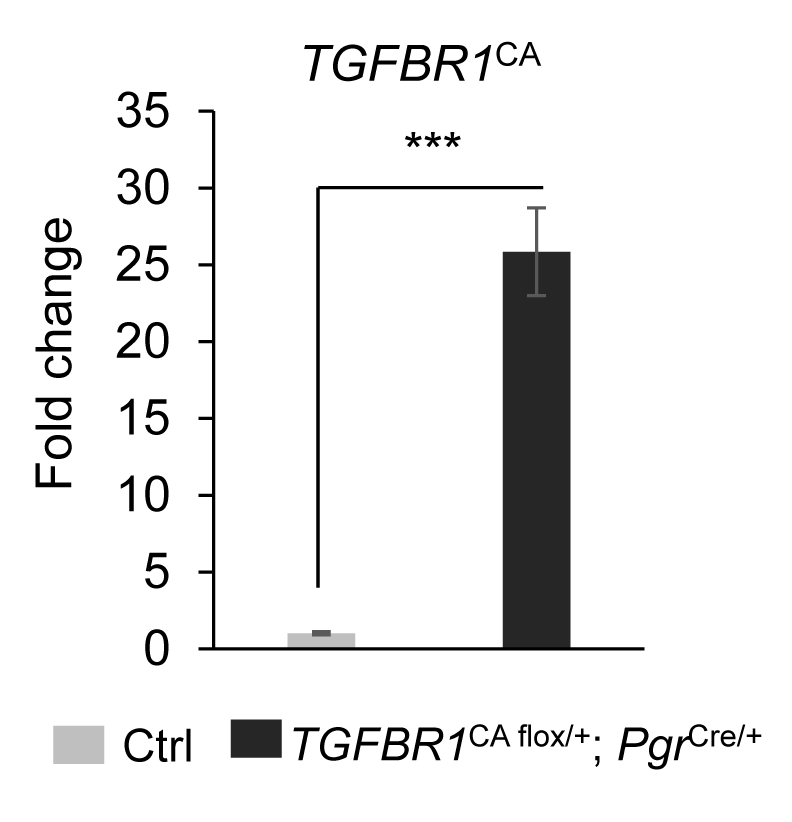

Supplement: S4 Fig — n = 4 for control and n = 6 for TGFBR1CA flox/+; PgrCre/+ mice. Rpl19 was used as internal control. Data are means ± SEM. ***P < 0.001. (TIF) [file pone.0209417.s004.tif]
